# Endoscopic management of esophageal mucosal bridges in children with esophageal atresia

**DOI:** 10.1007/s00464-023-10492-1

**Published:** 2023-10-13

**Authors:** Matthieu Antoine, Usha Krishnan, Michael Manfredi, Julija Cervinskiene, Jérôme Viala, Julia Brendel, Christos Tzivinikos, Audrey Vanrenterghem, Georges Dimitrov, Bruno Hauser, Noémie Laverdure, Barbara Rohmer, Hélène Behal, Audrey Nicolas, Frédéric Gottrand

**Affiliations:** 1 Division of Gastroenterology, Hepatology and Nutrition, Department of Paediatrics, University of Lille, Inserm, CHU Lille, U1286 - INFINITE, 59000 Lille, France; 2 Lille University Jeanne de Flandre Children’s Hospital, Avenue Eugène Avinée, 59000 Lille, France; 3 Pediatric Gastroenterology, Sydney Children’s Hospital, Sydney, Australia; 4 School of Women’s and Children’s Health, University of New South Wales, Sydney, Australia; 5 Gastroenterology, Hepatology & Nutrition, Boston Children’s Hospital, Boston, USA; 6 Endoscopy, Children’s Hospital, Vilnius University Hospital Santaros Klinikos, Vilnius, Lithuania; 7 Gastroentérologie Pédiatrique, Hôpital Universitaire Robert-Debré, AP-HP, Paris, France; 8 Klinik für Kinderchirurgie, Medizinische Hochschule Hannover, Hannover, Germany; 9 Pediatric Gastroenterology and Nutrition, Al Jalila Children’s Specialty Hospital, Dubai, United Arab Emirates; 10 Pédiatrie Médicale, CHU Amiens Picardie, Amiens, France; 11 Chirurgie Pédiatrique, CHR Orléans, Orléans, France; 12 Gastroentérologie, Hépatologie et Nutrition Pédiatrique, UZ Brussel, Brussels, Belgique; 13 Hépato-Gastroentérologie et Nutrition Pédiatrique, Hôpital Femme Mère Enfant, Lyon, Lyon, France; 14 ULR 2694 - METRICS: Évaluation des Technologies de Santé et des Pratiques Médicales, University of Lille, CHU Lille, 59000 Lille, France

**Keywords:** Esophageal atresia, Esophageal mucosal bridge, Pediatric gastrointestinal endoscopy

## Abstract

**Background and study aims:**

Esophageal mucosal bridge (EMB) may be diagnosed at the anastomotic site in children operated on for esophageal atresia (EA) but so far only a few cases (*n* = 4) have been reported. This study aimed to characterize EMB in children with EA, risk factors, and treatment.

**Patients and methods:**

This retrospective multicenter study recorded patient’s characteristics, EMB diagnosis circumstances, endoscopic management, follow-up, and EMB recurrence in children with EA aged less than 18 years, compared with paired EA patients without EMB.

**Results:**

Thirty patients were included (60% male, 90% EA/tracheoesophageal fistula, 43% associated malformations). Compared to 44 paired controls, EMB was associated with a history of nasogastric tube feeding (31% vs. 9.1%, *p* = 0.02) and severe gastroesophageal reflux disease (history of fundoplication: 41.4% vs. 13.6%, *p* < 0.01). 77% had symptoms (food impaction and/or dysphagia). Endoscopic management was performed in 53% of patients (83% electrocoagulation) with no technical difficulties or complications. 80% of the symptomatic patients with EMB improved after endoscopic treatment, independently of anastomotic stricture dilatation or not.

**Conclusion:**

EMB endoscopic management by electrocoagulation is safe and often leads to symptom improvement.

Esophageal atresia (EA) is a rare congenital malformation involving 1 in 2500 children [1]. Following surgery, EA patients often present digestive symptoms and dysphagia which can be caused by esophageal dysmotility, anastomotic strictures [2], gastroesophageal reflux disease (GERD) [3], and peptic and eosinophilic esophagitis [4]. Esophageal mucosal bridges (EMBs) (Fig. 1) are structures crossing the lumen of the esophagus. EMB is a rare entity, often resulting secondary to trauma from nasogastric tube, inflammation (Crohn’s disease, lupus), infections (HIV, HSV, esophageal candidosis, tuberculosis), radiation, and esophageal varices sclerotherapy [5]. Patients with EMB may be asymptomatic or may present with symptoms (chest pain, dysphagia). A few cases (*n* = 4) of EMB have been reported so far in children with EA [6, 7], and information about their role in symptoms and the need for treatment is lacking in this population.

**Fig. 1:**
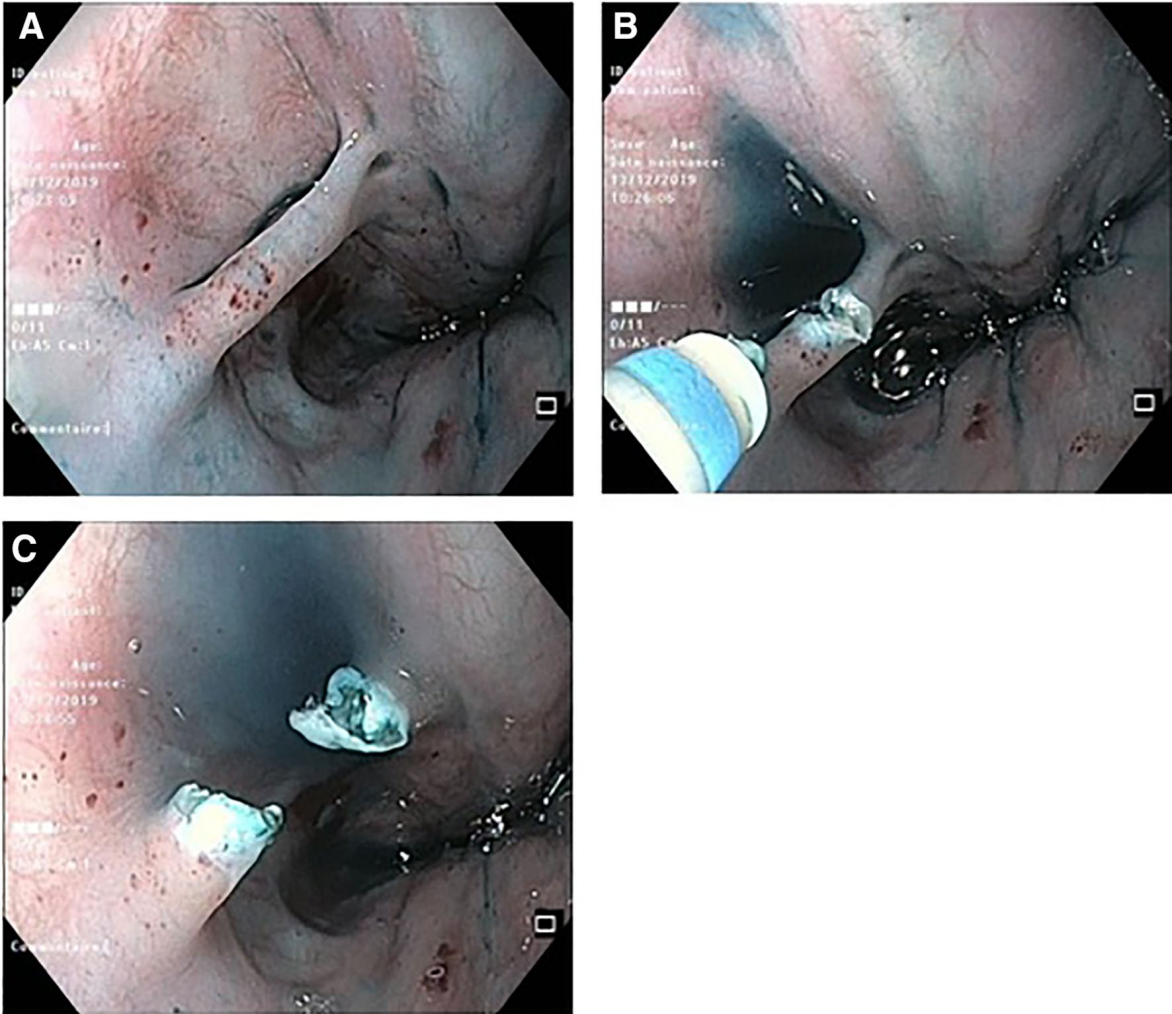
Esophageal mucosal bridge (**A**). Section by electrocoagulation (**B**), view after section (**C**)

We aimed to study diagnosis circumstances in a series of EMB in children with EA, identify risk factors of EMB, and assess the safety and efficiency of the endoscopic management of EMB.

## Patients/material and methods

The present study is a multicenter international retrospective study, including all reported children with EA < 18 years, in which EMB was identified at the site of esophageal anastomosis during esophagogastroduodenoscopy (EGD) between 1995 and 2022 in the participating centers. At least one control (EA patients without EMB) was selected for each case in each participating center. We also compared the characteristics of the studied population to a population-based EA registry [8].

### EMB cases

Patients were included if

1. History of EA surgery,
2. Presenting with EMB at EGD, and
3. Aged 0 to 18 years at the time of diagnosis of EMB.

An anonymized electronic standardized form was sent to expert physicians in charge of children with EA, using different networks (International Network on esophageal atresia [INoEA], European Rare Disease Reference Network [ERNICA], ESPGHAN EA working group, French national EA registry). Questions addressed the patient’s medical history and characteristics at the time of EMB diagnosis, EMB characteristics and management, and clinical and endoscopic follow-up.

The efficiency of the EMB management was only assessed in symptomatic children (i.e., with dysphagia and/or food impaction). We defined improvement when dysphagia or food blockages disappeared or reduced by more than 50% in frequency in the year following the EGD.

### Control

For each EMB case included, one to two control patients were required. Controls were patients with EA requiring an EGD who did not demonstrate an EMB, having similar age (± 1 year) and date of EGD (± 2 years) than EMB patients. Controls were paired on the co-existence of an anastomotic stricture (AS) or not.

### EA French register

To compare the gross characteristics of EMB cases with the general EA children population, we extracted data from the EA French register from 2008 to 2021 (*n* = 2199).

### Ethics

The study was conducted in accordance with protocols, good clinical practice, and the relevant laws and regulations in France. This study was approved by the Ethics Committee of the Gastroenterology, Hepatology, and Nutrition French Speaking Group (2020-029) as well as in every participant's ethical committee. Information letters and no-opposition forms were sent to the patient and their parents or legal guardians. The study was declared to the French Data Protection Authority (Commission Nationale Informatique et Libertés). International Review Board approval and written consent were not needed for this retrospective observational study. All data were anonymized.

### Statistical analysis

A logistic regression model was used to identify risk factors of EMB formation, with and without adjustment on AS. The clinical improvement was compared between groups using the Chi-square test or Fisher’s exact test. Statistical testing was done at the two-tailed α level of 0.05. Data were analyzed using the SAS software package, release 9.4 (SAS Institute, Cary, NC).

## Results

Thirty patients with EMB and 44 control patients were included from 11 centers (France, Lithuania, Germany, Belgium, Australia, USA, United Arab Emirates).

### Characteristics of children with an EMB

Sixty percent of the children (*n* = 18) with an EMB were male. EA Ladd classification was type III in 90% (*n* = 27) and type I in 10% (*n* = 3). EA was associated with other malformations in 43% (*n* = 13). All patients had a surgical anastomosis, at a median age of 1 day [1;2]. The mean age at EMB diagnosis was 5.5 ± 4.4 years old (range: 7 months–16 years). Patients with EMB had the same characteristics as the general population of EA patients (male gender: 58%, type III Ladd classification: 89%, associated malformations: 55%). EMB was localized at the anastomosis in 90% of the patients.

### Circumstances of diagnosis

EGD was performed for dysphagia (*n* = 21) ± bolus impaction (*n* = 5) in 77% of the cases, routine surveillance in six patients (systematic follow-up of EA), and various reasons in five patients (i.e., tracheoesophageal fistula follow-up, respiratory signs during meals). At the time of endoscopy, 11 children (36.7%) had an AS and 2 children (6.7%) had an eosinophilic esophagitis.

### Risk factors of EMB formation

A history of nasogastric tube and prior fundoplication were associated with EMB (EMB vs. control patients: 31.0% vs. 9.1%, *adjusted p* = 0.02; 41.4% vs. 13.6%, *adjusted p* = 0.01, respectively). No differences were found for the other factors we studied including GERD, history of anastomotic leak, history of peptic esophagitis, and AS (Table 1).

**Table 1:** Comparison of medical history of EMB patients to controls

	EMB patients		Control patients		*p*^a^	*p*^b^
	*N*	(%)	*N*	(%)		
Anastomotic leak	5/25	(20)	2/34	(5.9)	0.21	–
History of nasogastric tube feeding (excluding neonatal period)	9/30	(31)	4/44	(9.1)	0.02	0.02
Gastroesophageal reflux disease (GERD)						
History of GERD	25/30	(83.3)	32/44	(72.7)	0.29	0.36
History of positive pH-impedance metry	15/18	(83.3)	11/15	(73.3)	0.67	–
Current GERD	14/18	(77.8)	9/15	(60)	0.45	–
History of peptic esophagitis	11/30	(37.9)	9/44	(20.5)	0.1	0.1
Fundoplication	12/30	(41.4)	6/44	(13.6)	< 0.01	0.01
Esophageal dilations (ED)						
History of ED	19/30	(63.3)	23/44	(52.3)	0.35	0.24
Number of ED, median [IQR]	4 [2; 6]		3 [1; 5]		–	–

Statistical analysis for a history of pH-impedance metry and current GERD was made with patients in whom pH-impedance metry results were available

The numbers of patients for anastomotic leak analysis are different because the information was missing for a part of the patients

^a^Statistical analysis without adjustment

^b^Statistical analysis with adjustment on anastomotic stricture

### Prevalence of EMB and proportion of EMB with endoscopic treatment by center

The number of children with EMB during the inclusion period, the number of endoscopic treatments, and the number of children born with EA with surgical repair, by center, are shown in Table 2. The estimated prevalence of EMB was 1/72. The rate of endoscopic treatment performed in children with EMB varies largely between the centers.

**Table 2:** Number of EMB cases per center during the inclusion period (1995–2022) and EMB prevalence

Center	Number of children with EMB	Number of children with EMB with endoscopic treatment	Total number of children with EA repair
1	10	5	319
2	2	1	256
3	4	0	420
4	1	1	105
5	1	0	37
6	2	1	190
7	2	0	133
8	1	1	–
9	2	2	293
10	1	1	56
11	4	4	–
Total	30	16/30 (53.3%)	Total prevalence: 1/72

Prevalence was estimated from the centers in which we had the information on the total number of children born with EA with surgical repair during the inclusion period (1995–2022)

### EMB endoscopic management: technical aspects, safety

Sixteen patients (53.3%) underwent an endoscopic management of the EMB. Two recurred (12.5%) and underwent a second endoscopic management. Section of the EMB by electrocoagulation was mainly employed, in 15 EGD (tip cutting knife: 9, blunt tip knife: 2, unspecified: 4). Other techniques employed were argon plasma coagulation, hemostatic clip placement (to induce ischemia), and biopsy forceps in one patient each. For one patient, a hemostatic clip was used after electrocoagulation of the EMB for a preventive purpose. There was no complication or technical issue reported.

In the 11 children with associated anastomotic stricture, 15 esophageal dilatations were performed: 12 with hydrostatic balloons, and 3 with Savary-Gilliard bougies. The dilatation diameter was chosen in accordance to the age of the patient.

### Efficiency of endoscopic management in symptomatic children

Thirty patients with EMB were included. Twenty-three (77%) of them were symptomatic at the time the EGD was performed. They received 32 EGD: in 17 EGD (53.1%), no endoscopic treatment of the EMB was performed and only 4 children (23.5%) improved clinically. In 15 EGD (46.9%), there was an endoscopic treatment of the EMB: 12 (80%) were clinically improved (*p* < 0.001). The difference remained significant when we compared EMB cut only (8/10 improved: 80%) to the rest of the symptomatic patients in whom EMB was not sectioned or cut with associated dilatation of an AS (8/22: 36.7%) (*p* = 0.02) (Table 3).

**Table 3:** Efficiency of endoscopic management in symptomatic children

32 endoscopies in symptomatic children with mucosal bridge	Clinical improvement	*p*
	*n* (%)	
Section (± dilatation) (*n* = 15)	12 (80)	0.001
No section (*n* = 17)	4 (23.5)	
Only section (*n* = 10)	8 (80)	0.02
No section or section with dilatation (*n* = 22)	8 (36.7)	

Thirty patients with EMB were included. Twenty-three (77%) were symptomatic at the time of EGD. In these 23 children, 32 EGDs were performed

No significant difference in clinical improvement was found between children in whom an associated AS was dilated (7/15: 46.7%) and children with no associated AS (9/16: 56.3%), independently of an endoscopic section of an EMB cut or not (*p* = 0.59). Similarly, we found no difference when we compared children with AS dilatation only (3/10 clinically improved) with the other (AS dilatation with EMB cut, or EMB cut only, or no intervention: 13/21: 61.9%) (*p* = 0.14).

## Discussion

To the best of our knowledge, our study is the first and largest series of EMB in children with EA. It shows that EA should be added to the list of causes of EMB. Although it seems a rare complication of EA surgery (whose prevalence was estimated in our study at 1/72), our results show that EMB can cause symptoms—dysphagia and food blockage—in more than three-quarters of the children even if there is no associated AS.

We found that a history of nasogastric tube feeding and fundoplication were associated with EMB diagnosis. The pathophysiology of EMB remains unclear but usually involves trauma and inflammation [5]. The clear mechanisms of EMB genesis remain unknown. The nasogastric tube has already been reported as a cause of EMB formation [9]. Children with EA may need nasogastric tube feeding, in the perioperative period but also sometimes for nutritional support since undernutrition remains a concern in this population [10]. The prevalence of GERD is high in children with EA [3] and may be aggravated by esophageal dysmotility [11] which causes inflammation. The high prevalence of fundoplication in children with EMB that we found in our series may be an indicator that a serious GERD may induce EMB formation. A case of a 7-year-old boy with a refractory GERD requiring fundoplication diagnosed with an EMB was previously reported [12]. An anastomotic leak might be involved in the pathophysiology of EMB because it induces local inflammation. However, we could not find any significant difference in EMB according to a history of anastomotic leak in our patients. Another hypothesis is the suture of back-walling mucosa during the initial repair. This was however never reported in the esophagus or any other organ in association to mucosal bridge. Moreover, several children in our study had a normal endoscopy without EMB earlier in their medical history, suggesting that anastomosis surgical techniques could not be the only risk factors for EMB. Otherwise, this is the first time that the co-existence of eosinophilic esophagitis and mucosal bridge has been reported. Eosinophilic esophagitis can also cause inflammation and worsen dysmotility, and it may potentially be a risk factor for EMB, especially in EA patients.

Whether EMB is an incidental finding or can cause symptoms remains a matter of debate. Although non-interventional our study strongly suggests that EMB can be responsible for symptoms in EA patients: 77% of the EA patients presented with dysphagia and blockage and in 50% of them no other causes (i.e., AS or eosinophilic esophagitis) were found. The endoscopic section of the EMB led to clinical improvement in 80%, while only a minority of those with “conservative” EMB improved. There are a few cases of EMB reported in children in literature (*n* = 5) [6, 7, 12]. All were symptomatic and the mucosal bridge was cut in four of them, while it was not treated cut in one child. All the children with mucosal bridge management were improved. The only patient without endoscopic management of EMB remained dysphagic. Whether or not an EMB found incidentally in an asymptomatic patient should be sectioned remains an open question.

Endoscopic management of EMB was mainly (83.3%) electrocoagulation. Cutting EMB by electrocoagulation is safe in expert hands: there were no complications nor technical issues reported in our study. To the best of our knowledge, there is no previous case report of EMB cut by electrocoagulation. There are several cases of EMB management in children with argon plasma coagulation [6], hot biopsy forceps [12], and miniature stapler [7], with no complications reported. The usefulness of a clip in a non-vascularized lesion is questionable.

This study has both strengths and limitations. The strengths are the large number of cases of EMB in children with EA, with a comparison to paired children with EA but no EMB. The limitations are the retrospective and observational design of the study, which cannot prove causality.

In conclusion, in symptomatic children with EA and EMB, resection of EMB by electrocoagulation should be considered, as it is safe and results in symptomatic improvement, in addition to treatment of GERD, eosinophilic esophagitis, and AS.
